# Supplementary material for: Interactive molecular dynamics in virtual reality for accurate flexible protein-ligand docking
Source: PLoS One. 2020 Mar 11;15(3):e0228461. doi: 10.1371/journal.pone.0228461 (PMC7065745; doi:10.1371/journal.pone.0228461)
Supplement: S1 File — (PDF) [file pone.0228461.s001.pdf]

Supplementary Information

1. Data & Video files

The Open Science Framework (see the URL at http://doi.org/10.17605/OSF.IO/NCFQM) includes the raw simulation data utilized to generate all of the Figures in the main text and the SI, the associated Narupa input files, and the videos referred to within the text. The specific version of the Narupa software used to generate the simulations discussed in the text is available at https://irl.itch.io/narupaxr. The videos and animations discussed in the main text are also available on Vimeo at the following hyperlinks:

Supplementary Animation A: https://vimeo.com/354828618

Supplementary Animation B: https://vimeo.com/354829098

Supplementary Animation C: https://vimeo.com/354829412

Supplementary Video A.1: https://vimeo.com/354833443

Supplementary Video A.2: https://vimeo.com/380015176

Supplementary Video B: https://vimeo.com/354833800

Supplementary Video C: https://vimeo.com/354834013

2. System parameterization

Systems were selected where an existing crystal structure in PDB format exists. For trypsin and benzamidine, the bound complex coordinates were based on PDB file 1S0R. For HIV-1 protease and amprenavir, the bound complex coordinates were based on PDB file 1HPV. For neuraminidase and oseltamivir, the bound complex coordinates were based on the starting structures used in the production runs in previous work by Woods *et al.* (which were themselves based on PDB file 2QWK) [1]. For the user studies described in the main text, two additional ligand systems were identified: trypsin and indole-amidine, based on PDB file 2G5N; and neuraminidase and zanamivir, based on PDB file 6HCX.

For performing iMD-VR runs, all proteins were parameterized with the Amber 14ffSB force field[2]. Benzamidine, indole-amidine, zanamivir, and amprenavir were parameterized with GAFF using antechamber. If needed, hydrogens were added to the systems using reduce [3]. Parameters for oseltamivir were taken from Woods et al [1]. All three systems used the OBC2 implicit water model. All systems were energy minimized before being used as the starting coordinates for each bound complex. All iMD-VR simulations were run at 300K with a timestep of 0.5 fs. When interacting with atoms, we utilized a gaussian force, where the amount of force applied depends on the distance between the user's controller and the atom they were selecting. Once users stopped interacting with a selection, the velocities of all atoms in that selection were reinitialized according to a Boltzmann distribution based on the temperature in order to prevent any remaining interaction energy in molecules propelling them past where the user intends to place them. Further details of how the velocities are reset can be found in ref [4].

For the expert-level tests, an 800 kJ/mol/nm^2 restraint was added to all backbone atoms in both trypsin and neuraminidase; additionally, this restraint was applied to any Ca2+ cations embedded within both systems. An 800 kJ/mol/nm^2 was applied to all backbone atoms in HIV-1 protease, excluding those which make up the flaps which gate the active site (defined as residues 49 to 55 in chain A and residues 48 to 54 in chain B). A separate force was applied to the HIV-1 flap backbone atoms which, unlike the other backbone restraint, the user could turn on and off during the interactive simulation. The software default value of 2000 kJ/mol/nm2 force constant was used for these restraints. A higher force constant is used compared to the backbone restraints due to their interactive nature. When a user wishes atoms to be held in place, it is expected that they want to be held in place firmly. All training and testing non-expert user tasks for trypsin and hiv-1 protease had the same backbone restraints as the expert tests; these restraints were removed for the neuraminidase blind task for one two novice cohorts that were recruited. The other cohort used the same backbone restraints as the expert-level test. In future work, we intend to carry out a more detailed sensitivity analysis of different restraint regimes.

3. NarupaXR input files & settings

Input files for every system discussed in this work can be downloaded from the ESI. The input files consist of a PDB structure showing the starting bound coordinates, an .xml file which contains the simulation information to be loaded into NarupaXR, and a .json file

which will automatically render the molecules into the same representations used in this work. Files are compatible with NarupaXR version 2019.0.0; details on how to load these files into NarupaXR can be found in the version 2019.0.0 documentation.

4. Equilibration protocols

For three of the protein systems, a single frame (the minimum RMSD along the iMD-VR trajectory) was extracted from the simulation trajectories for production run molecular dynamics, whose results are shown in Fig 4. The extracted frame from trypsin had an RMSD of 0.041 for benzamidine compared to the starting coordinates. For neuraminidase the extracted frame had an RMSD of 0.27 for oseltamivir and 0.302 for the 150-loop compared to the starting coordinates. For HIV-1 protease, the extracted frame had an RMSD of 0.218 for amprenavir and 1.383 for the active site flaps compared to the starting coordinates.

Extracted frames were solvated and neutralized: 10515 water molecules and 9 Cl- ions were added to trypsin and benzamidine, 17956 water molecules and 1 Cl- ion were added to neuraminidase and oseltamivir, 10696 water molecules and 5 Cl- ions were added to HIV-1 protease and amprenavir. All water was treated with the TIP3P water model. Forcefields for the protein and ligand were kept consistent between the interactive simulation and production run.

The solvated frames were minimized and equilibrated using the following protocol. Post minimization, the system was linearly heated over 20 ps, starting at 30K and increasing the temperature by 30K every 2 ps, until a temperature of 298K was reached. Next, a 10 kcal/mol/$Å^2$ restraint was added to the protein backbone atoms and the simulation was run for five sets of 10ps under the NVT ensemble, reinitializing the velocities of atoms in the system before each stage. Next, any restraints on the protein were removed and the box volume was allowed to relax to a pressure of 1 bar by running under the NPT ensemble for 9 nanoseconds. Finally, the system was switched back to the NVT ensemble, before being run for a final nanosecond. We note that there are a number of ways which we could have chosen in order to initiate the subsequent 200 ns MD simulations, and it is unclear *a priori* which choice is optimal. We chose the minimum simply because it is uniquely defined and unambiguous. Given that the snapshots which we extracted for production molecular dynamics were first solvated, minimized and then equilibrated for 10ns, the minimum RMSD structure would have been immediately relaxed to other nearby fluctuating structures; it is therefore unlikely that choosing the minimum as the starting point gives a markedly different result than choosing some other average structure nearby.

The final box dimensions are as follows: For trypsin and benzamidine the dimensions were 69.109 by 66.786 by 75.289, for neuraminidase and oseltamivir the dimensions were 88.788 by 81.528 by 82.755, and for HIV-1 protease and amprenavir the final box dimensions were 68.894 by 65.427 by 78.456.

5. RMSD calculation

Narupa requires a set of atom coordinates from which to begin an iMD-VR simulation. These starting coordinates were used as the reference frame for the RMSD calculation. As such, the RMSD was calculated as a measure of the distance the ligand has moved compared to the starting frame (i.e. when it is in a bound position). To calculate the RMSD for the ligand, the protein backbone atoms in each step of the trajectory were aligned to those in the starting coordinates. Once the trajectory was aligned, the RMSD was taken for the ligand atoms compared to the starting coordinates (excluding hydrogens). However, an additional consideration is that all molecules tested display symmetry in some of their functional groups. Narupa allows users to quickly explore rotational orientations, which means that users can flip the orientation of certain rotationally invariant groups, and inadvertently introduce a degree of variation into RMSD calculations. Therefore, when calculating the RMSD of the ligand, symmetrical groups were identified (see figure S1) and the swap plugin of VMD was used to calculate a more optimal RMSD value.

Additionally, to give an idea of the RMSD when docked, a 'baseline' RMSD was calculated by allowing each system to run for 50 ps in Narupa without any perturbation from the user, using a timestep of 0.5 fs and recording interval of 0.25 ps, totalling 200 RMSD data points. In order to gain an idea of the native behaviour of each ligand within a fully flexible protein, the backbone restraints were relaxed to 5 kJ/mol/nm^2. In total, 60 ps of simulation time was recorded, however, the first 10 ps was discarded to give the simulation the opportunity to relax itself. Any ions in the system used backbone restraints of 800 kJ/mol/nm^2. As with above, the RMSD was taken excluding the position of any hydrogens in the ligand. The RMSD value was either taken as (a) a single mean value over this 'reference' trajectory or (b) a range, calculated as plus or minus two standard deviations from the mean. These values can be found in Table S2. A dataset containing the RMSD values for each reference trajectory of each system can be found in the supplementary files of the ESI.

6. NarupaXR technical specifications

Narupa consists of two components: A molecular dynamics server engine that propagates a simulation forward and transmits the atomistic data to a client application, which renders the simulation in VR, and provides the user interface. For small to moderate sized protein simulations, these two components can be run locally on the same machine. Alternatively, a server machine can run the simulation engine and transmit the atomistic data to other VR clients, allowing multiple people to interact with the same iMD-VR simulation. For the expert-level user tests, both the molecular dynamics engine and VR front end were run on a high-end Alienware 15 R4 gaming laptop with an Intel i7-8750H 2.20GHz CPU and a NVIDIA GeForce GTX 1070 graphics card. For non-expert user tests, both the molecular dynamics engine and VR front end were run on a high-end desktop PC with an AMD Ryzen 31200 3.10GHz CPU and a NVIDIA GeForce GTX 1060 GPU. During the novice user tests, an expert explained the protein system to the user in a multiplayer simulation, which required two **client** machines, with the data from the simulation engine transmitted to a computer with identical specifications over a local area network.

7. Participant-generated bound poses

Figure 6 in the main text shows an overlay of the minimum RMSD bound pose achieved by each of the five novice participants, showing the increased variation when participants had ghost atoms (top three images) and when they did not (bottom three images). Additionally, for the final 5 ps of the participant-generated trajectories in cohort 2, the distance between atoms in the ligand molecule with key atoms in the protein residue are shown in Figure S4. Table S1 shows the minimum RMSD of each participant for each task.

8. Generation of HIV-1 open flap coordinates

In order to successfully recreate the opening and closing process of HIV-1 protease, an indication of the correct open flap position was created by creating a ghost atom representation of the backbone atoms of open HIV-1 protease (PDB code: 1TW7) and then superimposing that structure over the HIV-1 protease starting coordinates. Next, an iMD-VR user took advantage of the three-dimensional capabilities of Narupa and manually inspected the 'hinge-point' of the two flaps, where the positions of backbone atoms in the open and closed positions start to diverge. From this, a trace atom representation was created with two sets of ghost atoms, one for the closed and one for the open position, giving users a sense for the normal range of movement of the flaps. The areas of the protein backbone encapsulated by the hinge points were defined as between residues 48 and 54 on both protein chains.

9. Docking and redocking without backbone restraints

After establishing the usability of our framework, the experts repeated the tests with the 800 kJ/mol/nm^2 backbone restraints removed from all simulation files. Experts were able to manipulate all three systems with minimal interference to the protein tertiary structure, indicating that backbone restraints can potentially be removed. However, an added benefit of using relatively high backbone restraints is that paths generated are more straightforward to interpret because the motion from thermal fluctuations is curtailed. As a result, PathReducer provides principle components which better capture the key features of user-sampled iMD-VR pathways (i.e. the movement of a ligand from a protein binding site rather than side chain fluctuations). For cases where loop displacement is important (e.g., the motion of residues in the 150-loop during the novice neuraminidase testing tasks), this can be a potential contributor to poor task accomplishment. For example, comparing the re-establishment of the 150-loop for the neuraminidase testing task where both backbone restraints are on and off, figure S5 shows that the RMSD of these key residues is poorer and more varied when backbone restraints are not present. Furthermore, Figure S4 shows that contact between the zanamavir carboxylate group and Arg371 (one of the trio of arginines which is important for binding) is consistently poor, indicating non-optimal placement of the ligand by participants - which is further supported by figure S3, which shows placement of the carboxylate group as better where backbone restraints are present. This would suggest that where placement of key residues is important, such as the 150-loop, backbone restraints have the benefit of restricting the degrees of freedom in a protein. For novice users who are unfamiliar with both iMD-VR controls and the protein system itself, a high backbone restraint gives users the ability to familiarize themselves with the process of undocking and redocking without fear of accidentally pushing the protein system into a state where binding is unfavorable.

10. PathReducer analysis

PathReducer takes as input an xyz file containing a series of molecular structures (in this case, an iMD-VR-generated trajectory) and uses principle component analysis to determine the optimal rotation and scaling of the original coordinate system that captures the most structural variance in the fewest coordinates. This simple rotation and scaling means that the resultant coordinate system is comprised of coordinates that are linear combinations of the input coordinates (or input 'features'). By looking at the coefficient (or 'weight') of each input coordinate in the new coordinate system, one can see the relative amount that input coordinate is contributing to the direction of the new coordinate. The projections of the original data onto the resultant coordinates are referred to as principal components (PCs). Plots of iMD-VR pathways in PC space provides an idea of the similarity of structures sampled along the pathway. These plots can also be used to understand the 'route' taken by a trajectory. Figure 3 of the main text shows the iMD-VR-generated binding and unbinding pathways in the space defined by their top two and top three PCs. The structures input into *PathReducer* were represented as squared interatomic distance matrices, owing to their rotational and translational invariance compared to Cartesian coordinates.

For computational tractability, the PCA considered only those 70,000 interatomic distances that varied the most along a trajectory (only taking into account non-hydrogen atoms). In the plots in Figure 3 of the main text, purple corresponds to the beginning of the trajectory and yellow corresponds to the end, with blue and green passed through in between. Each scatter point is a frame from the iMD-VR trajectory, and a linear interpolation was used to connect each point.

11. Demographic Information

Novice users were recruited in two cohorts of five, totalling ten participants total. Cohort one completed the testing and training tasks for trypsin and neuraminidase, where all protein systems had a restraint placed on their backbone atoms. Cohort two completed the testing and training tasks for trypsin, neuraminidase and HIV-1 protease, however, for the neuraminidase testing task, all restraints were turned off. For cohort one, of the five participants, three participants were female and one was male. One participant opted not to specify their gender. Four participants were between the ages of 18 and 24 and one was between the age of 25 and 34; one participant was an undergraduate taught student, one participant was a postgraduate taught student, and three were postgraduate research students. Prior experience with VR was low amongst the cohort: three participants rated themselves as having a little experience using VR and one participant rated themselves as somewhat experienced. One member of the cohort rated themselves as very experienced. For cohort two, of the five participants, four were male and one was female; two participants were between the ages of 18 and 24 and three were between the ages of 25 and 34; two participants were postdoctoral researchers, two were postgraduate research students and one was a postgraduate taught student. Prior experience with VR was low amongst the cohort: two participants rated themselves as having no experience using virtual reality, two participants rated themselves as being slightly experienced, and one participant rated themselves as somewhat experienced.

12. Additional figures and tables

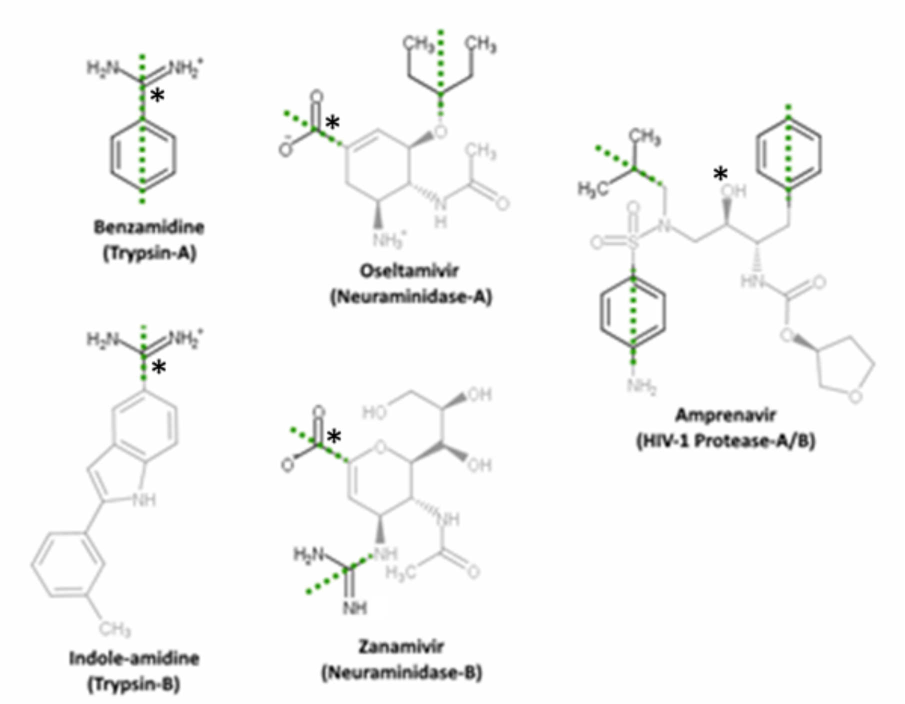

Figure S1 The symmetrical groups in each of the interactively undocked and redocked molecules. In these cases, the swap plugin of VMD was used so they could be treated as rotationally invariant when calculating RMSD. The atoms labelled with * are the key ligand atoms which participate in the distances shown in Fig S4.

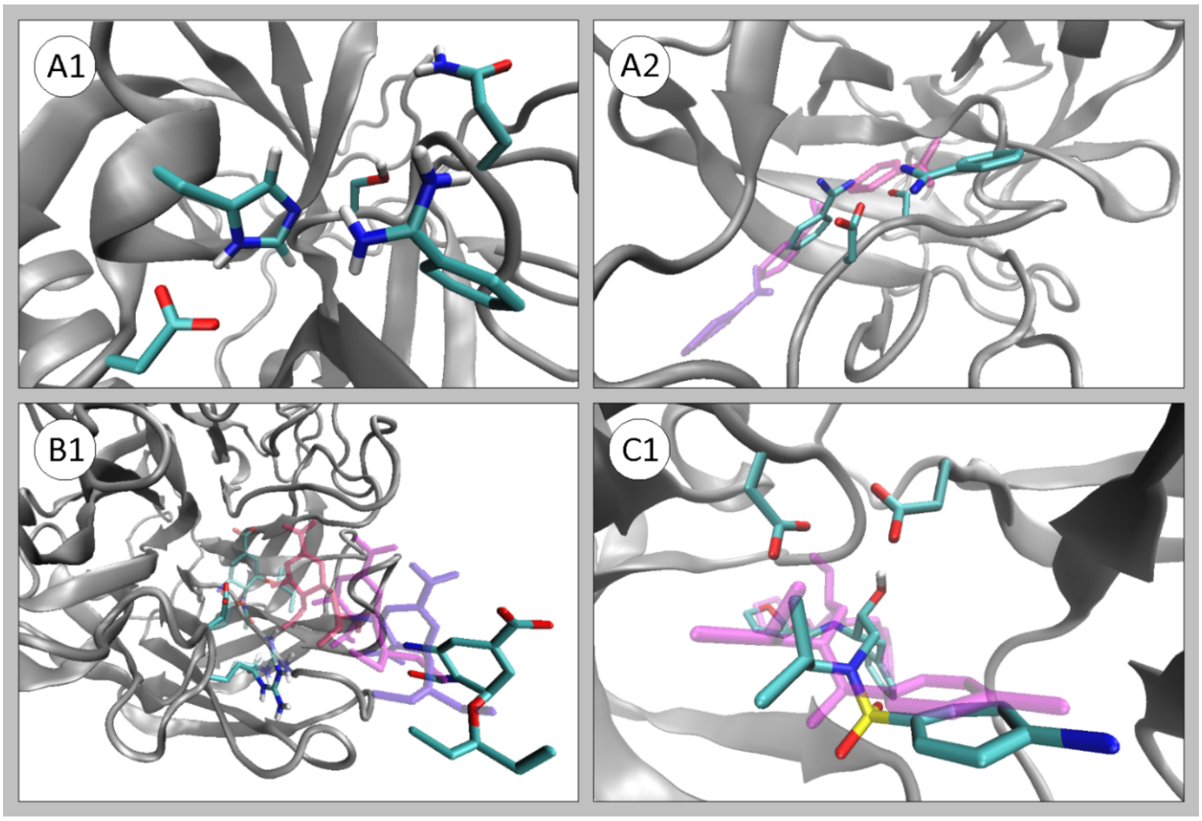

Figure S2 Snapshots of iMD-VR generated trajectories that illustrate observations by the expert level users. (A1) Shows the binding pose of incorrectly orientated benzamidine after being repelled from the trypsin binding pocket, in particular, how it binds to the catalytic Ser-His-Asp triad of benzamidine (plus neighbouring Glu192) on the way out, as shown in Supplementary video A.2. (A2) shows the path trypsin takes when being moved by an expert iMD-VR user through the hydrophobic protein core. In this case, starting from the docked pose, the user was able to pull benzamidine into a binding mode where it instead sits underneath Asp189 from this pose; the user was then able to continue pulling benzamidine until it exited the protein through an alternative pocket. Snapshots demonstrating the motion of the ligand are rendered as semi-transparent purple (B1) shows oseltamivir flipping its orientation when being pulled out of the neuraminidase binding pocket. Snapshots demonstrating the motion of the ligand oseltamivir as rendered as transparent purple; additionally, the initial positions of oseltamivir and the 150-loop are rendered as semi-transparent and in the CPK colouring style. (C1) shows an alternate binding pose of amprenavir into a tautomer of HIV-1 protease, where neither of the catalytic aspartic acid residues are protonated, demonstrating how the structure of amprenavir sags when the hydroxyl group switches which aspartic acid it points towards. The starting binding pose is rendered as semi-transparent and purple.

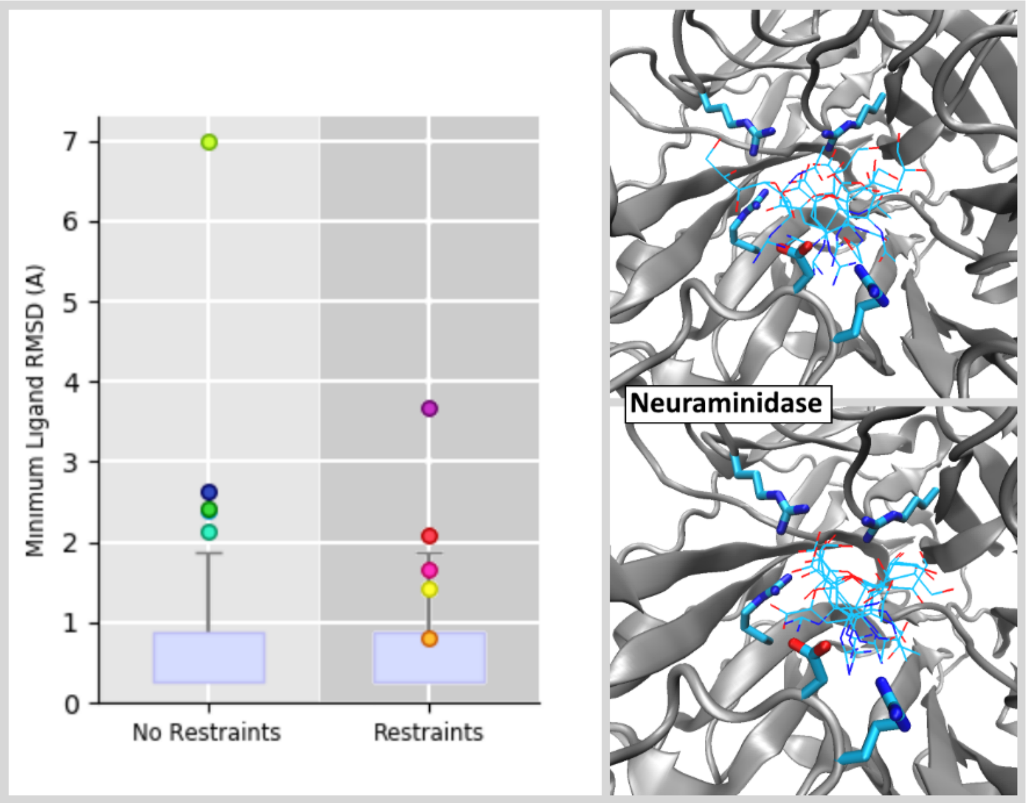

Figure S3 (Left) Minimum achieved RMSD for zanamivir in neuraminidase (testing task), where five participants completed the task without backbone restraints (cohort one) and with backbone restraints (cohort two). Each participant is assigned their own colour). (Right) Bound poses corresponding to minimum RMSDs shown on the left. No restraints is the top panel, restraints is the bottom panel.

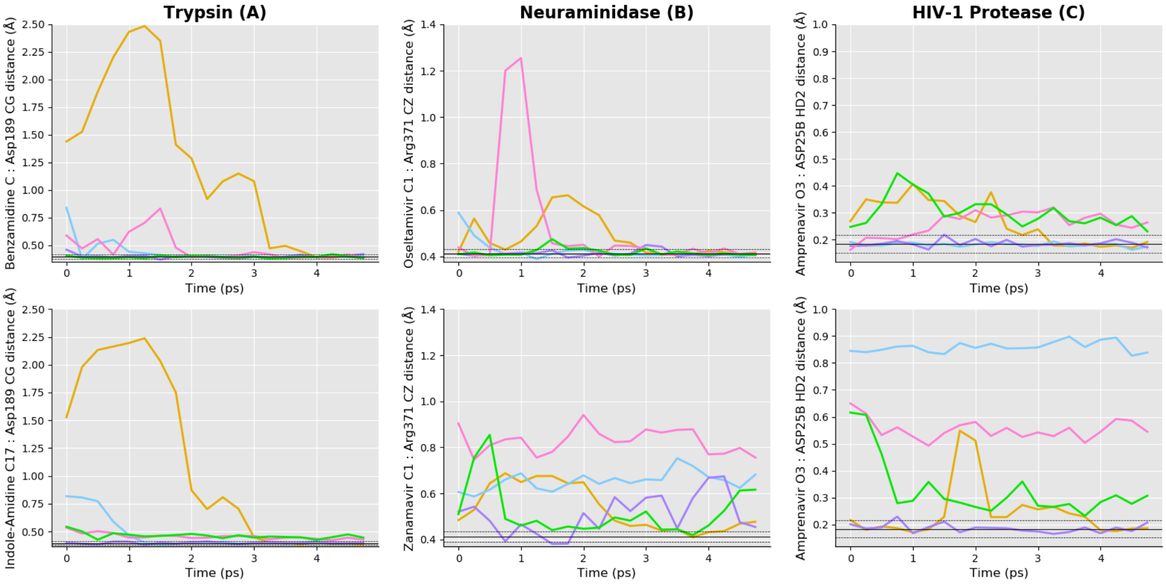

Figure S4: atom distances between key ligand atoms (labelled with * in Fig S1) and key protein residues (shown in figure 1 and 2 of the main text) through the final 5 ps of trajectories generated by 5 user study participants from cohort two, where each color represents the results generated by a different participant. The top row shows the results for training tasks, and the bottom row shows the results for testing tasks. The solid black line shows the system-specific average (with black dotted lines representing ±2σ) for a non-interactive Narupa simulation run for 50 ps in the bound pose and the calculated average atom distance is shown by the blue line. For benzamidine and indole-amidine, the distance represents the contact between their amidine group and Asp189 of trypsin. For oseltamivir and zanamavir, the distance represents the contact between their carboxylic acid group and Arg371. For amprenavir, the distance represents the contact between the hydroxyl oxygen and the protonated catalytic residue of HIV-1 protease, Asp25B.

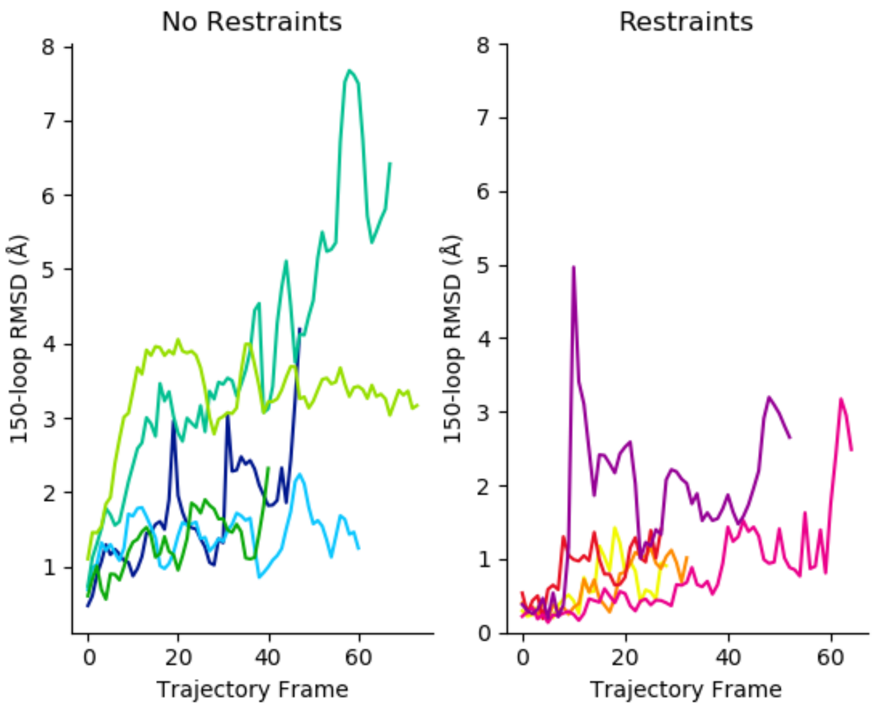

Figure S5 shows the RMSD of the two 150-loop residues, Asp151 and Arg152, of neuraminidase for the testing task, both with and without backbone restraints, showing that the RMSD of the 150-loop without restraints was (a) higher and (b) exhibited a greater degree of variation compared to with restraints. Each participant is assigned their own color.

Participant	Ligand RMSD (Å)						
	A training	A testing	B training	B testing (restraints)	B testing (no restraints)	C training	C testing
**C1 – 1**	0.23	2.61	0.27	1.41	*n/a*	*n/a*	*n/a*
**C1 – 2**	0.18	2.91	0.48	0.80	*n/a*	*n/a*	*n/a*
**C1 – 3**	1.13	1.49	0.64	2.08	*n/a*	*n/a*	*n/a*
**C1 – 4**	0.20	0.74	0.21	1.67	*n/a*	*n/a*	*n/a*
**C1 – 5**	0.23	1.53	0.48	3.68	*n/a*	*n/a*	*n/a*
**C2 – 1**	0.43	1.39	0.58	*n/a*	2.63	1.26	4.71
**C2 – 2**	0.16	1.20	0.73	*n/a*	2.40	0.85	6.82
**C2 – 3**	0.20	1.93	0.81	*n/a*	2.13	0.85	1.19
**C2 – 4**	1.15	2.20	0.74	*n/a*	2.42	1.14	1.04
**C2 – 5**	0.18	3.00	0.87	*n/a*	6.99	1.88	2.15

Table S1 The reported minimum ligand RMSD values for each participant for each task. Protein systems are categorized by letter, where A is trypsin, B is neuraminidase and C is HIV-1 protease. Participants are sorted by cohort, where C1 denotes the first cohort and C2 denotes the second cohort. Where a participant did not complete a given task, the RMSD value is n/a.

Participant	Ligand RMSD (Å)						
	A training	A testing	B training	B testing (restraints)	B testing (no restraints)	C training	C testing
**C1 – 1**	0.34	2.81	0.27	1.44	*n/a*	*n/a*	*n/a*
**C1 – 2**	0.38	3.29	1.22	1.12	*n/a*	*n/a*	*n/a*
**C1 – 3**	1.16	2.70	0.81	3.47	*n/a*	*n/a*	*n/a*
**C1 – 4**	0.50	1.02	0.52	2.47	*n/a*	*n/a*	*n/a*
**C1 – 5**	0.39	3.23	0.57	6.34	*n/a*	*n/a*	*n/a*
**C2 – 1**	0.47	2.35	0.58	*n/a*	3.22	1.86	5.20
**C2 – 2**	0.29	1.81	0.93	*n/a*	2.81	1.25	7.46
**C2 – 3**	0.62	1.93	0.91	*n/a*	7.59	1.23	1.24
**C2 – 4**	1.19	3.40	0.74	*n/a*	4.00	2.54	1.15
**C2 – 5**	0.24	3.33	1.20	*n/a*	8.92	2.16	2.55

Table S2 The reported final ligand RMSD values for each participant for each task. Protein systems are categorized by letter, where A is trypsin, B is neuraminidase and C is HIV-1 protease. Participants are sorted by cohort, where C1 denotes the first cohort and C2 denotes the second cohort. Where a participant did not complete a given task, the RMSD value is n/a.

System	Average RMSD (± 2σ) (Å)
**Benzamidine (A training)**	0.60 (± 0.30)
**Indole-Amidine (A testing)**	2.14 (± 1.23)
**Oseltamivir (B training)**	0.93 (± 0.47)
**Zanamavir (B testing)**	0.56 (± 0.31)
**Amprenavir (C testing and training)**	0.77 (± 0.40)

Table S3 The average RMSD of each ligand used in IMD-VR docking tasks, calculated over 50 ps of simulation time. Protein systems are categorized by letter, where A is trypsin, B is neuraminidase and C is HIV-1 protease. Two times the standard deviation is shown in brackets. Each value is given to three significant figures.

Supplementary Information References

1. Woods, C.J., et al., *Computational assay of H7N9 influenza neuraminidase reveals R292K mutation reduces drug binding affinity.* Sci Rep, 2013. **3**: p. 3561.
2. Maier, J.A., et al., *ff14SB: Improving the Accuracy of Protein Side Chain and Backbone Parameters from ff99SB.* J Chem Theory Comput, 2015. **11**(8): p. 3696-713.
3. Word, J.M., et al., *Asparagine and glutamine: using hydrogen atom contacts in the choice of side-chain amide orientation11Edited by J. Thornton.* Journal of Molecular Biology, 1999. **285**(4): p. 1735-1747.
4. O'Connor, M.B., et al., *Interactive molecular dynamics in virtual reality from quantum chemistry to drug binding: An open-source multi-person framework.* The Journal of Chemical Physics, 2019. **150**(22): p. 220901.
